# The American Medical Association

**Published:** 1888-02

**Authors:** 


### THE AMERICAN MEDICAL ASSOCIATION.

The next meeting of the American Medical Association is appointed for the second Tuesday in May, at Cincinnati.

Dr. Dawson, of that city, is the Chairman of the Committee of Arrangements. Any papers designed to be read before the Association, or any of its sections, should be sent, or a title indicative of its contents, and its length, to Dr. Dawson, the Chairman of the Committee of Arrangements, at least one month in advance of the meeting of the Association.

